# The Diversity Issue Revisited: International Students in Clinical Environment

**DOI:** 10.5402/2012/294138

**Published:** 2012-03-26

**Authors:** Marianne Pitkäjärvi, Elina Eriksson, Kaisu Pitkälä

**Affiliations:** ^1^Department of General Practice and Primary Health Care, University of Helsinki, Korpimaa 6 b 11, 02300 Espoo, Finland; ^2^Faculty of Health Care and Nursing, Helsinki Metropolia University of Applied Sciences, P.O. Box 4030, 00079 Metropolia, Finland; ^3^Unit of General Practice, Helsinki University Central Hospital, 00014 Helsinki, Finland; ^4^Department of General Practice and Primary Health Care, University of Helsinki, P.O. Box 20, 00014 Helsinki, Finland

## Abstract

*Background*. Globalization within higher education leads to an increase in cultural and linguistic diversity in student populations. The purpose of this study was to explore culturally diverse health care students' experiences in clinical environment in Finland, and to compare them with those of native Finnish students' participating in the same program. *Method*. A cross-sectional survey was performed at 10 polytechnic faculties of health care in Finland. 283 respondents (148 international and 95 Finnish students) responded to items concerning clinical rotation. The survey included items grouped as dimensions: (1) welcoming clinical environment, (2) unsupportive clinical environment, (3) approach to cultural diversity, (4) communication, and (5) structural arrangements. *Results*. International students felt as welcome on their placements as Finnish students. Concerning structural arrangements set up to facilitate preceptorship and approach to cultural diversity in the learning environment, the two groups' opinions were similar. However, international students were more likely than Finnish students to experience their clinical learning environment as unsupportive (*P* < 0.001). In addition, their experiences of communication with the staff was poorer than that of their Finnish peers' (*P* = 0.04). *Conclusions*. Awareness of strategies that enhance understanding, acceptance, and appreciation of cultural and linguistic diversity in any health care setting are needed.

## 1. Introduction

With the globalization of the higher education market, strong strategies and new innovations are needed to ensure that all students' needs and expectations are met. In order to better understand how learning of culturally diverse health care students could be facilitated the clinical environment, we investigated their experiences during clinical rotations.

Although the prerequisites of successful learning in the clinical environment among health care students are generally well known, factors associated with successful learning of culturally diverse students in the clinical settings are not equally understood.

In the context of clinical practice, the importance of staff's welcoming attitude was found to be associated with positive learning experiences for domestic students in their clinical rotation [1], for English-speaking students studying in another English-speaking country [2] and for students who lack domestic language proficiency [3, 4]. Both students [2, 4] and teachers [5] described how this manifested itself in staff's genuine interest in students' learning needs and cultural background. When staff members were friendly and willing to instruct, help, and support the students, they felt accepted, comfortable, and equal members of the staff [4, 5]. If, however, the staff's attitudes were unwelcoming, culturally or linguistically diverse students felt excluded, neglected, and lonely [4, 6, 7].

In unsupportive learning environment students were prevented from meaningful learning experiences and were assigned to low-level tasks instead [4, 6, 7]; in these situations students had to use observation as their primary method of learning. Under such circumstances students' behaviors varied: some chose to withdraw and to stay in the placement for the credits, only, whereas others kept on trying to make contact with staff and patients with considerable persistence [4]. Similar findings have been reported among international students [8–10] as well as among immigrant nurses [11, 12].

The significance of an orientation period for culturally or linguistically diverse health care students in clinical environment has been known for a decade [3, 13]; these studies showed that familiarizing students with patients, staff members, and facilities of the placement helped students becoming involved with diverse daily activities in the clinical environment. As described by Saarikoski et al. [14], Lekkas et al. [15], and Kell and Owen [16], several models exist for preceptorship in health professionals' education. Saarikoski et al. [14] concluded, however, that any type of preceptorship was significant for learning.

In explorations of students' perceptions of the difficulties they faced during clinical practice, literature suggested linguistic problems as a major obstacle for students who were not proficient in the domestic language(s) of the country in question. In this respect, findings from Australia [3, 13, 17, 18], the USA [19, 20], and Finland [4, 5], were similar. Use of complicated terminology, hospital slang, strong accent, and high speed when speaking, in particular, contributed to poor comprehension [13, 18]. Language barrier actualized itself in diverse clinical situations, such as in giving and receiving instructions, and understanding information during handover reports [18].

In addition to language-related difficulties, culturally diverse students and staff members also encountered discrimination, stereotyping, and racism in the clinical settings. Similar findings have been reported from the UK [6, 12], Finland [4, 5], the USA [10, 20], and Australia [11]. Rude behaviors could be expected from patients, staff members, fellow students, or coworkers [4].

There are scales available to measure the health care students' experiences in clinical settings [1, 21, 22], and since its publication, Saarikoski's [1] Clinical Learning Environment Scale (CLES) has been used in several countries. These scales, however, were not developed to acknowledge culturally diverse students' experiences, which, according to some prior studies [3, 4, 8–11, 19, 20], are unique.

To conclude, in spite of the wealth of the literature available about clinical practice, relatively few studies focus on culturally or linguistically diverse students' own experiences. To the best of our knowledge, the existing international evidence involving culturally diverse health care students in clinical environment is qualitative in nature.

Through a cross-sectional survey design, this study aimed at exploration of culturally diverse students' experiences in clinical environment in Finland. More specifically, the objective was to investigate students' background, life satisfaction, and experiences from clinical environment by comparing the international students' experiences with those of the native Finnish students' participating in the same program.

## 2. Materials and Methods

### 2.1. Population and Sample

**Figure 1:**
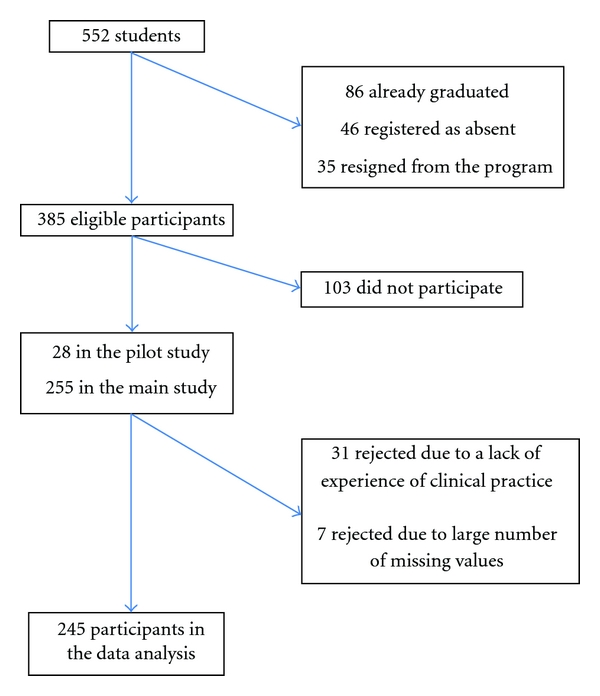
Flowchart illustrating the selection of the participants.

The population consisted of all students studying in the English-Language-Taught Degree Programs (ELTDP) in Finnish polytechnic faculties of health care during spring 2010. During the academic year 2009-2010, according to AMKOTA database owned by the Ministry of Education, there were 552 registered students studying in the degree program in nursing, public health nursing, and physiotherapy in the country. With the initial 282 participants, the response rate was 73.5%. Due to a lack of clinical experience, 31 students were excluded from this study and further 8 due to a lack of response to the items in this study. The selection of the participants is described in Figure 1.

### 2.2. Ethical Considerations

Initially, the ethical approval was obtained from the participating polytechnics. The purpose of the study and the voluntary nature of participation were explained to the students through an invitation letter. The data was collected anonymously, without revealing the identity of the participants or their respective polytechnics.

### 2.3. Questionnaire

In the questionnaire, age, gender, background education, length of residency in Finland, number of weekly employment hours, academic year, number of completed clinical practice rotations, self-reported English skills, self-reported Finnish or Swedish skills, and student experience of being satisfied with life during the past year were used as background variables.

As none of the existing scales [1, 21, 22] were developed to acknowledge culturally diverse students' unique experiences, a questionnaire comprising 27 items and 15 background questions was developed specifically for this study. To operationalize the student experience, we used the findings from qualitative studies [3, 4, 7–11, 20], including the earlier phases of our larger project [5]. All items concerning the students' experiences of clinical settings measured the degree of agreement through a modified Likert-scale with four classes (fully disagree-fully agree). Some of the items were in a positive form; others were in a negative form. The background information was examined through nominal or interval scale variables.

To ensure content validity, an expert panel was invited to comment on the questionnaire. The panel consisted of four experienced ELTDP-teachers from two schools. A draft of the questionnaire was distributed to the members of the panel and from the ensuing discussion a few items were reformulated and changed. To ensure face validity, eight ELTDP-students evaluated understandability of the items and clarity of the concepts. Their feedback resulted in minor modifications. Finally, the questionnaire was pilot-tested with 28 nursing students, and the questionnaire was found acceptable.

The five dimensions in the questionnaire were based on prior studies [3, 4, 7–11, 20], including some earlier stages of our research project [5]. Factor analysis was used to confirm these dimensions. The first one was called “Welcoming clinical environment”; items comprised a welcoming and friendly attitude, interest in supervision and willingness to work with the students, and finally, the students' experience of the quality of care and working atmosphere in the placement. The second one was called “Unsupportive clinical environment”; this was explored through the students' experience of being trusted, persistency needed in proving their competence, and, through feelings of loneliness, isolation, and being neglected. The third dimension, “Approach to cultural diversity in clinical environment,” comprised equality of the students, perception of the value of cultural diversity as well as its utilization, and acceptance in the clinical environment. “Communication in clinical environment” was investigated through aspects relating to the use of diverse languages. Finally, “Structural arrangements in clinical environment” were operationalized through availability of a preceptor and an orientation period, the ease of the students' involvement with the daily activities, the staff's awareness of the students' learning needs, and the clarity of expectations regarding students.

Internal consistency of the questionnaire was assessed with the Cronbach's alpha reliability coefficient. Alphas ranged between 0.78 and 0.90 for the different dimensions of the scale.

The sum variable for each dimension was created by dividing the total points by the number of questions the participant had answered.

### 2.4. Data Collection

The data were collected from all potential polytechnic faculties of health care (*N* = 10) during April 2010–January 2011. An ethical approval was obtained from the appropriate administrator at each of the ten polytechnics. The questionnaires were mailed to a contact person at each polytechnic. The contact person was a faculty member. The data collection was programmed to take place at school, either after the lessons or as part of the group tutorials. A letter of introduction explaining the purpose of the study, the voluntary nature of participation, and guaranteeing the confidentiality was attached as a cover page of the questionnaires. The questionnaires were returned in sealed envelopes.

### 2.5. Data Analysis

Data were analyzed using NCSS and SPSS version 15 (SPSS inc., Chicago, IL, USA). Descriptive statistics was used to characterize the sample. The dimensions were described by means and standard deviations.

To compare the characteristics and experiences of international and Finnish students, the *χ* ^2^-test and the Fischer exact test were used for categorical variables when appropriate and the Mann-Whitney *U*-test for nonnormally distributed continuous variable. The level of statistical significance was defined as <0.05.

## 3. Results

Of the 283 respondents, 148 international and 95 Finnish students responded items concerning opinions on clinical practice rotations (Table 1). Of the international respondents, 40.5% (*n* = 60) were from Africa, 30.4% (*n* = 45) from Australasia, 14.2% (*n* = 21) from Europe, and 6.1% (*n* = 9) from Central- or North America. The average age of all participants was 26.4 years. Of the respondents, 29% (*n* = 71) were first-year students, 31% (*n* = 76) were second-year student, 16.7% (*n* = 41) were third-year students, and 19.2% (*n* = 47) were fourth-year students. Over two-thirds (75.8%) of the respondents had been exposed to a minimum of two clinical practice periods.

As indicated by Table 1, compared to the Finnish students, the international students were older, more often males, and more often had a prior academic degree. The difference in the length of residency in Finland between the groups was significant. Observation of the native students' age and length of residency in Finland revealed that many of them had spent time abroad. Compared to their Finnish peers, the international students felt less often satisfied with their life. While investigating the possible link between life satisfaction and the dimensions among all participants, we found that those who were not satisfied with their life were more likely to experience their placement as unsupportive.

Comparison through the dimensions demonstrated that international students felt as welcome in their placement as Finnish students (*P* = 0.15) (Table 2). The international students' experience of staff's friendliness towards them was less positive than their peers' (*P* = 0.02), however.

The international students' experiences of unsupportive clinical environment were stronger than the Finnish students' (*P* < 0.001). A further exploration of the individual items within this dimension demonstrated statistically significant differences in international and Finnish students' feelings of loneliness (*P* < 0.001), not being trusted (*P* < 0.001), being ignored (*P* < 0.001), and feeling like an outsider (*P* < 0.001) during the clinical placements.

The two groups' opinions were similar regarding how cultural diversity was approached in the clinical environment. The item-by-item exploration indicated that the international students' experience of equality of students during the rotations was weaker than the Finns' (*P* = 0.015). Furthermore, the international students were more likely than the Finns to feel that the patients did not accept care provided by them (*P* = 0.043).

International and Finnish students' opinions differed with regard to their experiences of communication in the clinical environment (*P* = 0.04). An analysis of the individual items within this dimension pointed out that the international students did not feel the staff made an effort in trying to communicate with students without Finnish or Swedish proficiency (*P* = 0.004). Furthermore, they felt they were not approved by the staff due to weak Finnish or Swedish skills (*P* < 0.001). We also found that the language shift from English (theoretical instruction) into Finnish or Swedish (during placements), used up a lot of energy of the international students (*P* < 0.001).

The structural arrangements set up to facilitate preceptorship were found beneficial by both groups. Further analysis revealed, however, that the international students' experience of an orientation period in the beginning of the clinical rotation was less positive than their native peers' (*P* = 0.01). Additionally, compared to the Finnish students, the international students felt more often that they did not know what was expected of them (*P* = 0.003).

## 4. Discussion

Our findings show that although the international students felt welcome on their placements, they were more likely than the Finnish students to have experience of an unsupportive clinical environment. Compared to their Finnish peers in the same situation, the international students felt like outsiders who were ignored and not trusted. International students without fluent Finnish or Swedish skills also had negative experiences with communication during their placements.

The finding concerning unsupportive clinical environment suggested that the clinical component in health care students' education contains extra stressors for nonnative students. This was to be expected, as findings involving culturally or linguistically diverse students [7, 9, 10, 13] and professionals [11, 12] are similar around the world. A situation where any member of a health care team feels lonely, neglected or ignored by others is not compatible with the values of health professions or health care organizations.

Similarly to previous studies, communication in the clinical environment was found more challenging among the international students than among the Finns. Time and again a language barrier has been found to form the biggest obstacle in achieving positive outcomes for culturally diverse students during clinical rotations [3, 8, 10, 13]. By and large, the students [13, 18], the teachers [5], and the staff members [18] identify similar language-related challenges for the international students in any clinical environment. Examples include not knowing how to address patients appropriately [7, 18], or not being able to participate in clinical reasoning with other team members [3, 4, 18]. It was beyond the scope of this study to investigate which methods—other than language learning—could be utilized to ensure safe communication under circumstances where a language barrier exists between a student and a preceptor. Our findings, however, suggest that everyone involved with the process should pay particular attention to this issue.

Most students experienced their placement as a welcoming environment; a majority of the respondents also perceived the structural arrangements set up to facilitate preceptorship as positive. Such findings are encouraging, as they suggest that a number of organizations have successfully invested in developing their preceptorship practices.

Findings regarding clinical environments' approach to cultural diversity were mainly positive, although many—both international and Finnish students—felt diversity was neither utilized nor perceived as an asset. Instead of perceiving students' cultural diversity as enriching, Paterson et al. [9] found that the preceptors problematized cultural diversity of the students, as if it were less than the expected norm. A similar experience among both participant groups may explain some of our findings. Internationalization of the student population is a relatively new phenomenon in Finland and staff working in health care organizations might not know how to appropriately approach cultural diversity or to utilize it effectively.

The strength of this work lies in its participants: due to the relatively small number of potential participants, we chose to invite everyone who was enrolled as present during spring 2010. Thus, all ELTDP-s in Finnish polytechnic faculties of health care participated; the response rate may be considered good (73.5%). The careful development process of the questionnaire may be seen as another strength. Finally, for the first time, the methodology allowed us to compare international and native students' experiences in one specific field of study.

The limitations of the study relate to collecting the data by the local staff. This decision was mainly based on the long distances between the polytechnics, as well as on anticipated difficulties relating to reaching students at different stages of their studies in the same place at the same time. A relatively large number of participants did not identify their gender, age, or, country of origin, which may imply the respondents' concern for compromised anonymity. Also, a large number of the potential participants did not participate. This may have had to do with not being present during the data collection, not trusting that one's contribution would make a difference, or being concerned about anonymity.

The data were collected with a questionnaire specifically designed for this study. The items in this questionnaire were mainly generated from the literature. Such method of operationalization poses a threat to validity. Reliability of the measurement was examined by using the Cronbach's alpha reliability coefficient.

The participants of this study represented three different degree programs. Therefore, concerns may be raised regarding whether the questionnaire adequately acknowledges the unique characteristics of students and their respective programs.

## 5. Conclusions

There are still problems related to international students' preceptorship in clinical environment. Compared to their native peers in the same situation, the international students felt less supported, more often as outsiders and not being trusted to have the required competences. In addition to intensive language instruction for international students, awareness of strategies that enhance understanding, acceptance, and appreciation of cultural and linguistic diversity in any health care setting are needed.

## 6. Implications for Clinical Teaching

Strategies to support international students during clinical placements are to be developed. Tailored instruction which combines the subject matter and the language could form the core of such strategies. Further education for preceptors could comprise diversity issues, emotional effects of migration, and methods of communicating safely despite a language barrier.

## Figures and Tables

**Table 1:** Characteristics of international and finnish students participating in english language taught degree program at 10 polytechnic faculties of health care in Finland.

Variable	International students *n* = 148	Finnish students *n* = 95	*P* ^1^
Age, years			
Mean (range)	27.6 (20–48)	25.2 (19–53)	<0.001
Gender, male % (*n*)	33.8 (49)	11.7 (11)	<0.001
Resided in Finland, years			
Mean (range)	3.5 (0–16)	22.0 (1–42)	<0.001
Education, % (*n*)			<0.001
Upper secondary	47.7 (61)	81.1 (73)	
Vocational	16.4 (21)	11.1 (10)	
Academic degree	35.9 (46)	7.8 (7)	
Weekly working hours			
Mean (range)	17.7 (0–40)	11.3 (0–55)	<0.001
English skills, %, (*n*)			0.27
Basic or intermediate	18.0 (25)	23.9 (22)	
Advanced	82.0 (114)	76.1 (70)	
Finnish or Swedish skills, % (*n*)			<0.001
Basic	52.2 (70)	3.2 (3)	
Intermediate	35.1 (47)	3.2 (3)	
Advanced	12.7 (17)	93.6 (87)	
Feeling satisfied with life during the past year, % (*n*)			<0.001
Very often or often	57.0 (77)	84.0 (79)	
Occasionally or never	43.0 (58)	16.0 (15)	

^1^Differences between the groups were compared with *χ* ^2^ test or Fischer exact test in categorical variables and Mann-Whitney *U*-test for nonnormally distributed continuous variables.

**Table 2:** International and finnish health care students' experiences of clinical environment during rotations in Finland.

Variable	International students *n* = 148	Finnish students *n* = 95	*P* ^1^
Welcoming clinical environment			
Mean (SD)	3.32 (0.647)	3.43 (0.565)	0.15
Unsupportive clinical environment			
Mean (SD)	2.57 (0.704)	2.11 (0.522)	<0.001
Approach to cultural diversity in clinical environment			
Mean (SD)	3.06 (0.641)	3.02 (0.672)	0.70
Communication in clinical environment			
Mean (SD)	2.95 (0.707)	3.15 (0.526)	0.04
Structural arrangements in clinical environment			
Mean (SD)	3.19 (0.613)	3.19 (0.572)	0.87

^1^Differences between the groups were compared with the Mann-Whitney *U*-test.
